# Alcohol-Derived Acetaldehyde Exposure in the Oral Cavity

**DOI:** 10.3390/cancers10010020

**Published:** 2018-01-14

**Authors:** Alessia Stornetta, Valeria Guidolin, Silvia Balbo

**Affiliations:** 1 Masonic Cancer Center, University of Minnesota, Minneapolis, MN 55455, USA; storn005@umn.edu (A.S.); guido019@umn.edu (V.G.); 2 Division of Environmental Health Sciences, University of Minnesota, Minneapolis, MN 55455, USA

**Keywords:** acetaldehyde, alcohol, ethanol, oral cavity, cancer, exposure, microbiome, ALDH2, DNA adduct

## Abstract

Alcohol is classified by the International Agency for Research on Cancer (IARC) as a human carcinogen and its consumption has been associated to an increased risk of liver, breast, colorectum, and upper aerodigestive tract (UADT) cancers. Its mechanisms of carcinogenicity remain unclear and various hypotheses have been formulated depending on the target organ considered. In the case of UADT cancers, alcohol’s major metabolite acetaldehyde seems to play a crucial role. Acetaldehyde reacts with DNA inducing modifications, which, if not repaired, can result in mutations and lead to cancer development. Despite alcohol being mainly metabolized in the liver, several studies performed in humans found higher levels of acetaldehyde in saliva compared to those found in blood immediately after alcohol consumption. These results suggest that alcohol-derived acetaldehyde exposure may occur in the oral cavity independently from liver metabolism. This hypothesis is supported by our recent results showing the presence of acetaldehyde-related DNA modifications in oral cells of monkeys and humans exposed to alcohol, overall suggesting that the alcohol metabolism in the oral cavity is an independent cancer risk factor. This review article will focus on illustrating the factors modulating alcohol-derived acetaldehyde exposure and effects in the oral cavity.

## 1. Introduction

The oral cavity, as the initial section of both the digestive and respiratory systems and a site of exchange between the body and the external environment, is characterized by a high complexity of exposures. These exposures can result from exogenous sources such as air pollutants, tobacco, water contaminants, diet, and drugs or from endogenous processes such as cellular metabolism, inflammation, oxidative stress, and infections, as well as from chemicals derived from complex interaction with the oral microbiome [1,2,3]. Detrimental exposures in the oral cavity may lead to DNA damage that could result in mutations ultimately leading to the development of cancers of the upper aerodigestive tract (UADT) [4,5].

UADT cancers are an increasingly relevant public health issue, as they rank in the top six malignancies worldwide [6]. Epidemiological evidence indicates various factors being related to an increased risk of developing these cancers, including viral infections, poor oral hygiene, occupation (e.g., exposure to certain chemicals), diet, alcohol consumption, and tobacco smoking [1,6,7,8]. However, alcohol consumption and tobacco smoking are the factors that show the strongest association. For example, a study conducted in Northern Italy demonstrated that more than 80% of cancers of the oral cavity and pharynx are due to tobacco smoking and heavy alcohol consumption [9]. 

Tobacco has long been implicated as a traditional risk factor for UADT cancers, and the causal relationships between tobacco smoked, chewed or taken as snuff and cancer development are well established and reported [1]. UADT cancers are induced by the presence of carcinogens in tobacco. More than 7000 harmful chemicals were identified in tobacco and cigarette smoke or in its water-soluble components that will leach into saliva [10], and among them 70 were classified by the International Agency for Research on Cancer (IARC) as group 1 human carcinogens [11,12]. Among them, the potent carcinogenicity of polycyclic aromatic hydrocarbons and tobacco-specific nitrosamines have been studied extensively [13,14].

On the other hand, IARC classified ethanol as a human carcinogen to the oral cavity, pharynx, larynx, esophagus, liver, colorectum, and female breast [15,16,17], but the causal relationship between alcohol consumption and the elevated risk of cancer still remains unclear [18]. For UADT cancers, ethanol carcinogenicity seems to be mediated by acetaldehyde, its major oxidized metabolite. IARC has consequently classified acetaldehyde deriving from alcohol consumption as a carcinogen to humans [12,16,19]. Acetaldehyde induces DNA modifications that may result in mutations and cancer initiation [4,20]. 

Despite ethanol being metabolized mainly in the liver [21], some studies in humans have found higher levels of acetaldehyde in saliva compared to those found in the blood immediately after alcohol consumption [22,23,24]. In addition, increasing alcohol intake results in immediate increased acetaldehyde levels in the saliva, and high salivary acetaldehyde concentrations (50–100 µM) can be detected after ingestion of 0.5 g alcohol/kg body weight, an amount that can be reached by drinking about half a bottle of wine [25,26]. These findings suggest that ethanol-derived acetaldehyde exposure may occur in the oral cavity independently from liver metabolism, and that ethanol metabolism in the oral cavity may be considered a separate cancer risk factor. This hypothesis is supported by studies showing the presence and increased levels of acetaldehyde-derived DNA damage in oral cells of humans and oral tissues of primates when exposed to alcohol [27,28].

Alcohol-derived acetaldehyde exposure in the oral cavity can be influenced by several factors such as, metabolism of oral mucosa cells and salivary glands, including deficiencies of enzymes taking part in ethanol metabolism, metabolism resulting from the oral microbiome, poor oral hygiene, as well as nutritional, psychosocial, and environmental factors (e.g., type of alcoholic beverage consumed, tobacco smoking) [3,22,23,25,29,30,31,32,33,34,35,36,37,38,39,40,41]. Acetaldehyde is naturally present in some alcoholic beverages due to its production by yeasts, acetic acid bacteria, and by coupled auto-oxidation of ethanol and phenolic compounds [42]. An extensive epidemiological study suggested that naturally present acetaldehyde poses a cancer risk for drinkers of alcoholic beverages in addition to the risk given by ethanol-derived acetaldehyde [41]. On the other hand, acetaldehyde production and/or detoxification from the oral microbiome has opened a new research question about the possible link between microorganisms and oral cancer due to the high variety and number of microorganisms populating the mouth [3,25,43].

A clear identification of the various factors influencing acetaldehyde exposure and assessment of their importance in increasing acetaldehyde levels in the oral cavity may provide crucial information to better understand the key events characterizing alcohol-induced carcinogenesis in the UADT. This article will cover this topic by discussing all factors that can influence ethanol-derived oral acetaldehyde exposure (with a particular focus on the microbiome), and review ethanol pharmacokinetics, metabolism, as well as acetaldehyde-induced DNA damage and its biological consequences.

## 2. Ethanol Pharmacokinetics and Metabolism

### 2.1. Ethanol Pharmacokinetics 

The most common route of ethanol exposure is through ingestion of alcoholic beverages. The systemic pharmacokinetics of ethanol is strongly influenced by its physical-chemical properties. In fact, ethanol is a small polar amphipathic molecule with both lipophilic and hydrophilic characteristics [44], which is rapidly absorbed through passive diffusion by the stomach and by the upper small intestine [44,45]. The absorption rate is strongly affected by several factors such as the presence of food in the gastrointestinal (GI) tract, food type, and type of alcoholic beverage ingested [46]. Due to first pass metabolism, the bioavailability of ethanol is not 100%, but 90% [47]. Some ethanol may be oxidized by alcohol dehydrogenase (ADH) in the stomach, but chronic alcohol administration, sex, and the presence of food can strongly influence the impact of the first pass metabolism on the overall ethanol bioavailability [47].

As a result of its hydrophilicity, the volume of distribution of ethanol is close to the total body water volume, which is approximately 70% of body mass [48]. The ability of ethanol to hydrogen bond with water makes it highly water soluble allowing for its distribution in all water compartments of the body until an equilibrium between blood ethanol and tissue ethanol concentrations is reached [44,45]. As a consequence, the same dose of ethanol per body weight results in a different blood concentration depending on the individual fat and water proportions in the body [21]. Maximum blood alcohol concentrations are usually achieved approximately 75 min post consumption, but factors such as sex, age, and diet may contribute to variations in this amount of time [49].

**Figure 1:**
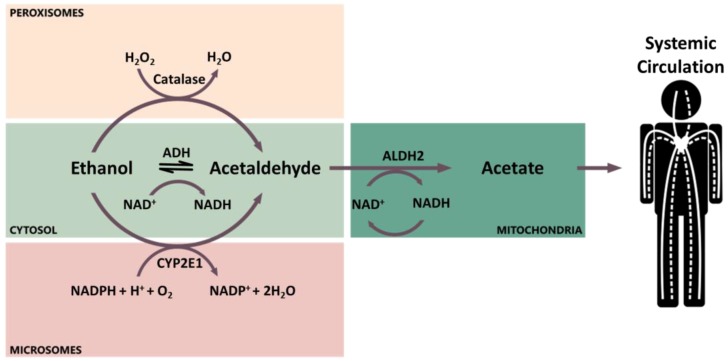
Schematic representation of ethanol metabolism in different cell locations.

A small portion (about 5%) of the absorbed alcohol is excreted unmodified in urine, breath, sweat, feces, milk, and saliva. The major portion is metabolized through phase I metabolism by three enzymes: alcohol dehydrogenase (ADH), aldehyde dehydrogenase (ALDH), and cytochrome P450 2E1 (CYP2E1, Figure 1) [48]. The primary site of ethanol metabolism is the cytoplasm of hepatocytes, where ethanol is oxidized by ADH into its major metabolite acetaldehyde [45]. Alternatively, ethanol can be oxidized into acetaldehyde in the smooth endoplasmic reticulum by the inducible enzyme CYP2E1, which is involved in the microsomal ethanol-oxidizing system [45]. However, the induction of CYP2E1 is reached only after prolonged heavy alcohol intake [45]. In addition, a small amount of ethanol is oxidized by catalase in the peroxisomes [50]. In the mitochondria, acetaldehyde is then oxidized by ALDHs into acetate, which can be released into the systemic circulation (Figure 1) [45].

Depending on the concentration of ethanol, different enzymes are involved in its elimination. The pharmacokinetics of ethanol is a combination of zero-order, first-order, and Michaelis-Menten kinetics. At low concentrations, ADH is primarily responsible for metabolism and ethanol elimination will follow Michaelis-Menten kinetics [51,52]. At high ethanol concentrations ADH is saturated, but due to the presence of CYP2E1 and some ADH isozymes zero- or first-order kinetics can be observed [48,53]. Ethanol is metabolically removed from the body at an approximate rate of 1 g/h [21]. The pharmacokinetics and detoxification rate of ethanol can change if there are differences in the expression of enzymes involved in ethanol metabolism, or due to ethnicity, sex, drugs, and individual genetic background [21].

### 2.2. Genetic Variants and Enzyme Induction Influencing Ethanol Metabolism

Genetic differences in the alleles encoding for ADH and ALDH enzymes may be associated to an increased production and decreased degradation of acetaldehyde, respectively. A highly active ADH converts ethanol into acetaldehyde more quickly, meanwhile, an inactive or less active ALDH avoids or decreases the conversion of acetaldehyde into acetate [20,54]. Differences in the activity of ADH and/or ALDH may therefore result into an accumulation of acetaldehyde [29,55]. On the other hand, even if polymorphisms for CYP2E1 are reported, the relationship between the variants and acetaldehyde production has still to be clarified. Since CYP2E1 is involved in ethanol oxidation into acetaldehyde, it may be hypothesized that a more active variant of the enzyme would lead to an increased acetaldehyde production from ethanol [20]. 

ADH enzymes are encoded by seven different genes, two of which, ADH1B and ADH1C, are polymorphic and thus exist in more than one variant. This polymorphism may result in differences in ADH activity and therefore ethanol metabolism [4,20]. For example, ADH1B has two alleles, ADH1B*1 and ADH1B*2, the second of which results into an enzyme form that is 40-fold more active than the enzyme form resulting from the first allele [4,20]. The ADH1B*2 allele is commonly found in East Asians and leads to a faster elimination of ethanol and accumulation of acetaldehyde [56]. In Caucasians, the more common ADH1C*1 allele encodes for highly active ADH enzymes, which lead to an increase in ethanol metabolism by about 2.5-times compared to ADH1C*2 [39]. 

Studies on the effect of ADH1C polymorphisms on the risk of developing UADT cancers have shown contradictory results. In a first case-control study in alcohol-dependent subjects in France, Coutelle and colleagues highlighted that the ADH1C*1 genotype was related to an increased risk of developing laryngeal and oropharyngeal cancers [57]. Coutelle’s findings were confirmed by Harty and colleagues through a case-control study conducted in subjects from Puerto Rico. In fact, it was reported that individuals homozygous for the ADH1C*1 allele, which consumed a high amount of alcohol, had a 5.3-fold increased risk of oral and pharyngeal cancer compared to ADH1C*1/2 and ADH1C*2/2 subjects [55]. Finally, in a third case-control study, an association was found between the ADH1C*1 genotype and the prevalence of alcohol-related head and neck cancer [58]. On the other hand, Boutchardy and colleagues did not find any association between the ADH1C*1 genotype and UADT cancers in Caucasian alcohol consumers [59]. This lack of correlation was also confirmed in another case-control study, in which subjects with head and neck cancer did not report any specific prevalence of an ADH genotype [60]. 

The relationship between ADH2 polymorphism and head and neck cancers was evaluated in a case-control study, in which the DNA of 34 Japanese alcohol-dependent men, who had one or more cancers of the aerodigestive tract, was genotyped [34]. It was found that the presence of the ADH1B*2 allele significantly increases the risk of oropharyngolaryngeal cancers, with an overall 6.68 odds ratio for subjects with the ADH1B*2 polymorphism in comparison with a 1 odds ratio for subjects with the ADH1B*1 polymorphism. The study also calculated individual odds ratios of 5.48 and 6.57 for oropharyngeal and laryngeal cancers, respectively. Meanwhile, the coexistence of ADH2 and ALDH2 polymorphisms showed to have a synergistic effect on the risk of oropharyngolaryngeal cancers, with an overall odds ratio of 121.77 [34]. 

ALDH2 is the enzyme mainly responsible for the detoxification of acetaldehyde. A very low activity of ALDH2 affects 40–50% of East Asians, due to a single nucleotide polymorphism (SNP) G-A in the coding region of ALDH2, which introduces a glutamine instead of a lysine at position 487. This mutation results in an increased exposure to acetaldehyde [61]. ALDH2*1 is considered the normal allele, whereas ALDH2*2 is the inactive variant. ALDH is a homotetrameric enzyme, thus individuals with ALDH2*1/2*2 have only the 6.25% of normal ALDH2*1 protein. ALDH2*2/2*2 homozygous and ALDH2*1/2*2 heterozygous have respectively an average blood acetaldehyde concentration 18- and five-times higher than the average of active ALDH2*1/2*1 homozygotes, when drinking a moderate dose of alcohol (0.8 g/kg body weight) [54]. The distribution of ALDH2*2 allele varies by race, and it affects 600 million people of East Asia, but is extremely rare in Caucasians and Africans [62]. 

Deficiency in the ALDH2 gene (ALDH2*2 allele) is associated with an increased risk of cancers of the digestive tract, both in alcohol-dependent and non-alcohol-dependent drinkers [17,33,63,64]. When drinking alcohol, the upper digestive tract mucosa of ALDH2-deficient subjects is exposed via saliva to about two times and via gastric juice to about five-to-six-times higher acetaldehyde concentrations than in subjects without mutations in the ALDH2 gene [22,29,30,56,65]. The relationship between ALDH2 genotype and the increased cancer risk was supported by a molecular initiating event investigation of the adverse outcome that revealed that alcohol-dependent patients with the ALDH2*1/2*1 allele did not have the DNA modifications detected in the blood of alcohol-dependent patients with the ALDH2*1/2*2 [66]. The large number of epidemiological studies underlying this relationship make subjects with the ALDH2 genotype a solid and unique cancer model for the evaluation of acetaldehyde toxicity [38]. 

For CYP2E1, inter-individual variations in its activity have been detected [67]. The human CYP2E1 gene is located on the long arm of chromosome 10, has a total of nine exons, and is characterized by several polymorphisms [68,69]. Two of the most studied polymorphisms of CYP2E1 are the wild allele CYP2E1*5A characterized by *RsaI* restriction site at position −1259 and CYP2E1*5B, which has a *PstI* restriction site at position −1019. As these sites are both located in the 5′-flanking region, they could play a role in the transcription of the CYP2E1 gene [70]. As a result, two alleles have been detected, the wild-type homozygous c1/c1, the wild-type heterozygous c1/c2, and the variant homozygous c2/c2 [68,71]. For the CYP2E1*5B genotype, the rare c2 allele is more frequent amongst the Eastern Asian population relative to Caucasians [72,73,74]. This polymorphism has been shown to result in higher transcription and increased activity of CYP2E1 [68], which may possibly lead to inter-individual differences in ethanol metabolism. Another polymorphism is found on the intron 6 and is designated as the *DraI* polymorphism CYP2E1*6 [75]. This variant has been shown to enhance transcription of the CYP2E1 gene [76], but it seems not to be associated with an increased expression or activity of the enzyme [77]. This polymorphism is prevalent in Caucasians, although at a lower frequency than that observed in Eastern Asian populations [78,79,80]. 

CYP2E1 can be induced in the liver, but also in other tissues, by chronic alcohol consumption [67]. This induction was found to be related to an increase in protein stabilization due to alcohol intake, rather than to an enhanced synthesis of the protein [81,82,83]. Lieber and colleagues first demonstrated the existence of a methanol oxidizing system able to adapt itself on ethanol feeding by evaluating ethanol metabolism in animal and human tissues [84]. A strong confirmation of this finding was reported by Oneta and colleagues after administration of 40 g of ethanol (corresponding to three regular drinks) per day for four weeks to five healthy man, and subsequent measurement of CYP2E1 activity by the chlorzoxazone test [85]. Results from this study showed an increase in the activity of CYP2E1 already starting from the first week of ethanol administration [85]. However, they also noticed that CYP2E1 activity decreased significantly three and eight days following ethanol withdrawal [85]. An induction in CYP2E1 may translate into more conversion of ethanol into acetaldehyde, and therefore an increase in acetaldehyde exposure that could potentially play a role in ethanol-mediated carcinogenesis in heavy drinkers.

Also for CYP2E1, the associations between polymorphisms and UADT cancers lead to contradictory results. There seems to be an association between wild-type c1/c1 genotype carriers of CYP2E1*5B and the risk of cancers of the oral cavity, larynx, esophagus, liver, lung, and pharynx compared to the variant genotypes [86]. On the other hand, an increased risk of cancers of the gastric, nasopharyngeal, hepatocellular, lung, and oral cavity was observed for the carriers of the variant alleles [86]. Ruwali et al. found a significant increase in the risk of developing head and neck squamous cell carcinoma (HNSCC) in subjects carrying the CYP2E1*5B and CYP2E1*6 polymorphisms [87]. The same study also found alcohol or tobacco use to interact with the genotypes in significantly enhancing the risk of developing HNSCC [87]. 

Bouchardy and colleagues showed that heavy drinkers had the highest risk of developing cancers of the oral cavity or pharynge, with a 7.2-fold increased risk for carriers of the c2 genotype, and a 2.5 times increased risk for those carrying the c1 genotype compared to light drinkers [59]. However, this interaction analysis was difficult to perform due to the low frequency of carriers of the CYP2E1 variants [59]. Gattas and colleagues found that the frequency of the CYP2E1 *PstI* allele was higher in patients with head and neck cancer compared to controls, but this association was found only for cancers of the oral cavity [88]. Hung et al. performed a case-control study looking for the interactions between oral cancer risk factors (cigarette smoking, alcohol drinking, and betel quid chewing) and genetic polymorphisms of CYP2E1, and they found that the c1/c2 and c2/c2 genotypes were associated with an increased oral cancer risk compared to the c1/c1 genotype among the subjects who did not chew betel quid, but not among chewers [89].

On the other hand, Katoh and colleagues did not find significant differences in the polymorphisms of CYP2E1 genes in 92 Japanese cancer patients and 147 unrelated non-cancer controls [90]. Similarly, Yang and colleagues assessed the influence of the CYP2E1 c2 variant allele on the risk of esophageal cancer in conjunction with alcohol consumption in 165 diagnosed Japanese subjects and 495 randomly selected controls [91]. Results did not show any significant alteration in the risk of developing esophageal cancer in respect to the CYP2E1 polymorphism [91]. Finally, a meta-analysis by Guo and colleagues investigated the associations between CYP2E1 *Rsal*/*PstI* polymorphisms and the risk of developing oral cancer [92]. This study found instead a significant association between these polymorphisms and oral cancer risk for c1/c1 versus c1/c2, but not for the model c1/c1 versus c1/c2 + c2/c2, concluding that these polymorphisms represent a risk factor for developing cancer of the oral cavity [92]. 

Traditionally, alcohol metabolism has been mainly associated with liver metabolism, and the consequences of heavy drinking with the development of liver cancer [20]. This belief would imply individuals with ALDH/ADH polymorphisms exposed to alcohol and heavy drinkers with CYP2E1 polymorphism to have an increased risk of developing cancer of the liver. However, a study by Yokoyama and colleagues showed no increase in the risk of developing liver cancer in ALDH2 deficient subjects [33]. Meanwhile, a significant increase in the risk of developing oropharyngolaryngeal, esophageal, stomach, colon, and lung cancers was found in the same subjects [33]. These findings suggest that for evaluating the relationship between alcohol and cancer, local metabolism in the organ or body part of interest has to be considered independently from liver metabolism. 

## 3. Factors Influencing Acetaldehyde Exposure in the Oral Cavity 

### 3.1. Genetic Polymorphisms 

Acetaldehyde levels in the oral cavity may vary depending on the individual genotype coding for ethanol-metabolizing enzymes (Figure 1). ADH and ALDH are primarily responsible for the amount of acetaldehyde present, whereas CYP2E1 contributes to up to 30% of the overall ethanol metabolism only in chronic alcohol-dependent subjects [21,93]. The effect of ALDH2 deficiency in the salivary level of acetaldehyde has been measured in several independent studies, with or without exposure to alcohol, to investigate whether acetaldehyde in the oral cavity can act as local carcinogen [22,29,30,38,65,94].

The first study reporting salivary acetaldehyde levels in ALDH2-deficient subjects found that the seven subjects that were heterozygous for the ALDH2*2 allele had two to three times higher salivary acetaldehyde levels, throughout the entire follow-up period of 240 min, than those (13 subjects) with normal ALDH2 after receiving a moderate dose of alcohol of 0.5 g/kg body weight [29]. This increase in acetaldehyde in the heterozygous population studied was found to be related to the inability of their parotid glands to metabolize acetaldehyde to acetate. However, this study pointed out the importance of assessing the contribution of the oral mucosa cell and/or submandibular parotid gland metabolism, which could also influence salivary acetaldehyde production [29]. 

The role of oral mucosa cells and/or submandibular parotid glands in salivary acetaldehyde production was tested in a related study, in which the higher salivary acetaldehyde level observed in ALDH2-deficient subjects was counteracted by 4-methylpyrazole (4-MP), a drug used for treating methanol and ethylene glycol poisoning as well as for reducing the flushing and blood acetaldehyde levels in ALDH2-deficient subjects. 4-MP decreases the rate of ethanol elimination by a competitive inhibition of the oxidation of ethanol to acetaldehyde by ADH [30,95]. When 4-MP was orally administered prior to ethanol exposure, the salivary production of acetaldehyde was significantly reduced in ALDH2-deficient participants, but not in normal ALDH2 volunteers [30]. Since 4-MP is more effective in inhibiting human than bacterial ADHs, the results supported the hypothesis that, in normal ALDH2 volunteers, the production of salivary acetaldehyde comes mainly from microbial origin than from metabolism of oral mucosa and glandular ADHs [30]. However, the study underlined the need to consider, in addition to the ALDH2 genotype, also other factors that may contribute to salivary acetaldehyde production from ethanol such as ADH genotype, smoking, drinking habits, and individual differences in the oral microbiome [30]. 

Another study measuring salivary acetaldehyde levels twice with a 1 h interval in intoxicated Japanese alcohol-dependent men found that acetaldehyde was higher in the saliva of ALDH2*1/*2 than in that of the ALDH2*1/*1 group, providing a possible mechanistic explanation for the increased risk for UADT cancers in this sub-population [56]. The link between ALDH2-deficient subjects and an increased risk of UADT cancers is observed in drinkers [33], possibly due to the increased exposure to acetaldehyde, but not among ALDH2 deficient non- or rare drinkers independently from their smoking habits or diet [96]. Therefore, the effect of the ALDH2 genotype on the salivary acetaldehyde levels was investigated also in subjects who did not ingest ethanol, but were locally exposed by it by rinsing their mouth with an ethanol solution (40% vol) [94]. Interestingly, the study found that after a brief mouth rinsing with ethanol, the salivary acetaldehyde concentration (measured at different time intervals up to 20 minutes after ethanol discharge) in ALDH2 deficient subjects did not differ from that of subjects with normal ALDH2 [94]. However, the authors noted that two minutes after consumption, averaged salivary acetaldehyde concentrations between the two groups (11 vs. 6) showed a near-significant difference (*p* = 0.06), which may become significant when increasing the number of subjects [94]. 

Effects of SNPs were investigated also for ADH 1B and 1C. ADH1C genotype in heavy alcohol consumers with and without UADT cancers was investigated, and results demonstrated that heavy drinkers homozygous for the ADH1C*1 allele have a predisposition to develop UADT cancers, possibly due to an increase in the levels of salivary acetaldehyde after consuming alcohol [31]. In fact, salivary acetaldehyde concentrations were found to be modulated by ADH1C genotype, with subjects homozygous in the ADH1C*1 allele having higher salivary acetaldehyde levels than heterozygous subjects [31]. The effect of ADH1B, ADH1C, and ALDH2 genotype on salivary acetaldehyde was assessed in alcohol-dependent men the morning after drinking [32]. Both persistence and level of salivary acetaldehyde were found to be higher in the saliva of alcohol-dependent subjects with the less active ADH1B*1/*1 than in the saliva of the alcohol-dependent subjects carrying the ADH1B*2 allele [32], and this finding may explain the increased risk for UADT cancers in this sub-population. 

There is clear evidence that genetic polymorphisms of ALDH and ADH enzymes are associated to a modulation of acetaldehyde exposure in the oral cavity. Subjects drinking alcohol exhibiting genotypes that result in less efficient or deficient ALDH are exposed to higher acetaldehyde levels than drinkers with active ALDH. Even if a link between salivary acetaldehyde and oral cancer has only been hypothesized, it is important to identify subjects with ALDH/ADH polymorphisms through genetic testing and to make them aware of the potential risks related to alcohol consumption and high acetaldehyde exposure in the oral cavity. On the other hand, there is currently no knowledge of the role of CYP2E1 polymorphisms on acetaldehyde levels in the oral cavity, and further investigation is needed to cover this gap.

### 3.2. Poor Oral Hygiene

The link between poor oral hygiene and levels of microbial-derived acetaldehyde in the oral cavity has been investigated, but only in a few studies. The role of dental health status on acetaldehyde production from ethanol in saliva was investigated in 132 volunteers. Poor dental status assessed by a measurable score derived from factors such as dentition, tooth brush frequency, denture wear, tooth loss, and frequencies of oral infections and sores was associated with a two fold increase in the salivary acetaldehyde production from ethanol in vitro by exposing saliva to ethanol for 90 min, underlying the possible role of dental hygiene and status in the risk of developing oral cancer in association with ethanol consumption [36]. Most of acetaldehyde in saliva comes from microbial metabolism [25], therefore, these findings suggest that the correlation between poor dental status and increased acetaldehyde levels may result from changes in the oral microflora (e.g., bacterial overgrowth or growth of super acetaldehyde-producing bacteria) caused by poor oral hygiene [36]. 

Another study investigated if poor dental health and oral hygiene could influence in vitro salivary acetaldehyde production in oral cancer patients and healthy volunteers [97]. For this purpose, 66 individuals were divided in three groups: oral cancer patients (group 1), poor dental health status (group 2), and good dental health status (group 3). Saliva samples were collected from all subjects and incubated in vitro with ethanol to assess salivary acetaldehyde production by head space gas chromatography [97]. Salivary acetaldehyde production was significantly higher in group 1 and 2 compared to group 3, underlying the role played by poor dental health, poor oral hygiene, and infrequent dental appointments on the levels of salivary acetaldehyde produced in vitro, and suggesting a possible link between salivary acetaldehyde production and oral cancer [97]. However, this study was limited by lacking information about the dental health and oral hygiene status of the cancer patients in group 1 [97]. Nevertheless, there seem to be a correlation between poor oral hygiene and increased salivary acetaldehyde, as hypothesized previously. 

### 3.3. Nutritional and Environmental Factors 

Several nutritional and environmental factors are able to modulate ethanol-derived acetaldehyde exposure and levels in the oral cavity. These factors can be divided in those increasing acetaldehyde levels such as smoking [37], type of alcoholic beverages [22,23,40,41,98,99,100,101,102], or alcohol-containing mouthwashes [103], and those decreasing the levels of acetaldehyde in the oral cavity such as the use of L-cysteine tablets [65,104], or of an antiseptic mouthwash [25]. In addition to ethanol-derived acetaldehyde exposure, the level of acetaldehyde in the oral cavity could also be modulated by acetaldehyde naturally present in food and non-alcoholic beverages [40,102,105], however, this review article focuses on alcohol-derived acetaldehyde exposure, and therefore these studies are not further discussed. 

The risk of developing UADT cancers is highly increased when alcohol drinking and tobacco smoking are combined, due to a synergistic and multiplicative effect [106,107,108,109,110]. In order to investigate whether this synergy is related to an increased exposure to acetaldehyde, the synergistic effect of alcohol consumption and smoking on salivary acetaldehyde was measured in seven smokers and six non-smokers [37]. For the ethanol-induced salivary acetaldehyde baseline, subjects ingested 0.8 g/kg body weight ethanol, and acetaldehyde in saliva samples was analyzed every 20 min for 160 min. Three days after the ethanol test, the smokers were again exposed to the same amount of ethanol, but this time samples were collected while they smoked one cigarette every 20 min for 160 min [37]. Results showed that (1) smokers had an ethanol-induced salivary acetaldehyde baseline two times higher than non-smokers after ethanol exposure, and (2) smoking during the ethanol challenge increased the salivary acetaldehyde levels in smokers compared to non-smokers by 7-fold [37]. The higher salivary acetaldehyde levels in smokers vs. non-smokers after ethanol exposure only has been hypothesized to be related to changes in smokers oral microbiome, or to the inhibition by smoking of ALDH in oral mucosa cells [37], whereas the 7-fold increased salivary acetaldehyde in smokers could be attributed to acetaldehyde present in cigarette smoke and to some synergistic and multiplicative effects. 

Another factor leading to an increased acetaldehyde exposure in the oral cavity is the presence, in some alcoholic beverages, of acetaldehyde as a result of alcohol fermentation. The level of acetaldehyde initially present in alcoholic beverages has been measured by gas chromatography or enzymatic techniques in a variety of samples [22,23,40,98,99,100,101,102], and results from those studies are summarized in Table 1. Values reported in Table 1 show that acetaldehyde not resulting from ethanol metabolism is present in a variety of alcoholic beverages and exhibits a big range of concentrations. Results are consistent among the different studies, with alcoholic beverages such as gin, vodka, and beer containing less acetaldehyde than wine and other types of spirits [22,23,40,100,101,102].

In order to verify if a high concentration of naturally present acetaldehyde also resulted in an increased acetaldehyde exposure in the oral cavity, three of the studies reported in Table 1 also measured salivary acetaldehyde levels in volunteers after drinking alcoholic beverages with varying naturally present acetaldehyde amounts [22,23,98]. For example, the study by Yokoyama et al. found that the salivary acetaldehyde concentration immediately after drinking wine was significantly lower than after drinking Calvados or shochu, demonstrating how intrinsic amounts of acetaldehyde in the drink influence the immediate acetaldehyde exposure [22]. In another study by Lachenmeier et al. subjects asked to rinse their mouth with an alcoholic beverage for 30 s had increased salivary acetaldehyde levels relative to the baseline 30 s after mouth rinsing, and concentrations of up to 1000 µM were reached when rinsing the mouth with grape mark spirit, the beverage with the highest naturally present acetaldehyde concentration measured [23]. 

Finally, Linderborg et al. measured salivary acetaldehyde levels in eight volunteers after tasting 5 mL of 40% vol ethanol with no acetaldehyde and Calvados (40% vol) containing 2400 µM acetaldehyde [98]. Results were consistent with previous studies [22,23], with the salivary acetaldehyde concentration being significantly higher 30 s after tasting Calvados than ethanol, but not significantly different two minutes after tasting [98]. Therefore, they concluded that naturally present acetaldehyde has a short-term effect in the acetaldehyde exposure in the oral cavity, and that the initial acetaldehyde concentration in the beverage increases salivary acetaldehyde level seconds immediately after drinking, but seems to become less relevant minutes after alcohol exposure, where metabolic acetaldehyde formation seems to be more prevalent [98]. However, consistency in the measuring time may be an issue in these studies and a more standardized universal way for evaluating acetaldehyde naturally present vs. acetaldehyde derived from ethanol exposure should be implemented. For example, a quantitative assessment via gas or liquid chromatography/mass spectrometry [40,111] of the initial acetaldehyde levels in the beverages to be administered should always be performed prior to exposure to derive the portion of acetaldehyde naturally present vs. that of acetaldehyde derived from individual ethanol metabolism.

Ethanol is not only present in alcoholic beverages, but it is also a constituent of some ready-to-use mouthwashes, in a concentration that can vary from 5% to 27% vol [112,113,114]. An increased oral cancer risk for users of ethanol-containing mouthwash has been hypothesized, however, epidemiological evidence has been inconclusive [115]. It is possible for the ethanol in the mouthwash to be metabolized to acetaldehyde by microbes populating the oral cavity of users. However, one of the functions of ethanol in mouthwashes is bacterial elimination, its use should therefore result in fewer microorganisms in the mouth that are able to metabolize ethanol into acetaldehyde and in a decrease of acetaldehyde exposure. 

Lachenmeier et al. investigated salivary acetaldehyde levels after the use of ethanol-containing mouthwashes and mouth rinses according to manufacturer’s procedure in healthy, non-smoking volunteers [103]. Saliva samples were collected at 0.5, 2, 5, and 10 min after rinsing of the mouth with the mouthwashes and mouth rinses. Despite the study having only four participants, the concentrations of acetaldehyde in saliva were significantly above endogenous levels, and comparable to those found after consumption of an alcoholic beverage [103]. On the other hand, Homann et al. demonstrated a reduction in the in vivo acetaldehyde production after a 3-day use of the antiseptic mouthwash chlorhexidine, which contains about 11.6% alcohol [25]. This reduction in salivary acetaldehyde levels may be caused by a decrease in the abundance of microorganisms in the oral cavity. In fact, Homann et al. found a significant decrease in baseline aerobic and anaerobic bacterial counts after chlorhexidine rinsing [25].

Among other factors influencing acetaldehyde exposure in the oral cavity, the administration of L-cysteine tablets has been proven to be successful in decreasing salivary acetaldehyde levels upon ingestion of ethanol [65,104]. Cysteine has the ability to covalently bind to acetaldehyde resulting in the formation of the stable and non-toxic 2-methylthiazolidine-4-carboxilic acid [116], in which the reactivity of acetaldehyde is trapped by the thiol group of the amino acid preventing reaction with proteins and DNA [104]. 

The ability of L-cysteine to decrease acetaldehyde exposure in the oral cavity was investigated in a panel of nine healthy male volunteers, who fastened a tablet of L-cysteine under the upper lip prior to ingestion of 0.8 g/kg body weight of 10% (*v/v*) ethanol [104]. Saliva samples were collected every 20 min for a total of 320 min. Results of this study were that salivary acetaldehyde concentration in the presence of L-cysteine was reduced by 59% compared to the placebo tablet, and that after alcohol intake up to two-thirds of acetaldehyde could be removed from saliva by slow and continuous release of L-cysteine [104]. L-Cysteine is classified by the European Food Safety Administration (EFSA) and the US Food and Drug Administration (FDA) as “generally regarded as safe” (GRAS) and is widely used as food additive [117,118]. Therefore, results from this study demonstrate how L-cysteine could be used to decrease acetaldehyde exposure in the oral cavity.

### 3.4. Other Factors

The factors discussed above are among the most studied known contributors of acetaldehyde modulation in the oral cavity. Additionally, due to the highly reactive nature of acetaldehyde, influences of its reactions with other biological molecules present in saliva or gingival crevicular fluid such as constituents of phase II metabolism (e.g., glutathione) should be considered as factors that may modulate acetaldehyde’s local concentration. For example, there is evidence of acetaldehyde being able to conjugate in vitro with cysteinylglycine, the first metabolite in the glutathione breakdown [119], suggesting that such a conjugation could decrease the level of free acetaldehyde in the oral cavity. Studies performed in animal models found decreased glutathione levels in gingival tissues of rats [120] and oral tissues of mice [121] after chronic alcohol consumption, further suggesting that the levels of acetaldehyde in the oral cavity may be influenced by glutathione and potentially by other components of phase II metabolism. Nevertheless, since little is known about the reactivity of acetaldehyde with such molecules and human studies are currently lacking, the influence of phase II metabolism on acetaldehyde levels in the oral cavity can only be hypothesized, and further investigation is needed to elucidate their impact. 

## 4. Ethanol Metabolism by Oral Mucosa Cells, Salivary Glands, and Oral Microbiome

During alcohol challenges, a major contribution of acetaldehyde exposure in the oral cavity is given by ethanol metabolism by the oral mucosa cells, salivary glands, but most importantly by the oral microbiome. In individuals carrying the normal ALDH and ADH alleles, the production of salivary acetaldehyde has been shown to come mainly from microbial origin than from metabolism of oral mucosa and glandular cells [25,30]. In addition, ADH activity of human oral mucosa is very low [122]. Finally, it has been shown that mouth rinsing with an antibacterial solution prior to alcohol exposure reduces salivary microbial counts and acetaldehyde levels by about 50% [25], confirming the major role of the oral bacterial microbiome in acetaldehyde exposure in the oral cavity. The relative contribution of ALDH activity from oral cells and the microbiome has not been systematically compared and investigated. Therefore, this section will mostly focus on the contribution of acetaldehyde exposure by oral microbiome metabolism. 

Due to their ADH activity, several microbes that are constituting a healthy oral microflora can oxidize ethanol into acetaldehyde [123,124], which can then accumulate into saliva. In fact, microbial capacity as well as that of oral mucosa cells to metabolize acetaldehyde into acetate is limited [123,125,126]. Interestingly, salivary acetaldehyde levels increase linearly when the alcohol dose is increased, since microbial ADHs cannot be saturated with ethanol [25,123]. However, ADH activity vary between the different oral microbial strains [127,128]. Table 2 summarizes oral microbial strains of yeasts and bacteria that have been shown to be able to oxidize ethanol into acetaldehyde in vitro.

Of the yeasts able to metabolize ethanol into acetaldehyde there is so far positive evidence only from *Candida* species (Table 2). Tillonen and co-workers first investigated the contribution of oral yeasts to acetaldehyde production in the oral cavity in 55 saliva samples that were divided in two groups based on their high and low in vitro ability to produce acetaldehyde from ethanol [132]. Yeast colonization was higher in the high (78%) compared to the low (47%) acetaldehyde producing saliva group, and the main isolated *Candida albicans* species differed in their acetaldehyde production capabilities, identifying *C. albicans* as a key player in microbially-derived acetaldehyde production in the oral cavity and underlying individual’s capacity to produce salivary acetaldehyde [132]. In another study, the ability of non-*C. albicans* species to produce acetaldehyde was investigated in vitro in 30 non-*C. albicans* isolates incubated with ethanol and glucose [131]. All 30 non-*C. albicans* isolates investigated produced potentially carcinogenic amounts of acetaldehyde. Among them, *C. tropicalis* showed the highest (252.3 µM), whereas *C. krusei* showed the lowest (54.6 µM) amounts of acetaldehyde produced [131]. Two of the yeasts isolated (*C. tropicalis* and *C. parapsilosis*) produced a higher amount of acetaldehyde from ethanol, 252.3 and 243.3 µM respectively, compared to the previously studied *C. albicans* (235.1 µM), underlying the importance of these two non-*C. albicans* species in the evaluation of microbially produced acetaldehyde exposure in the oral cavity [131].

In a study to determine which oral bacterial strains possess ADH activity and capacity to produce acetaldehyde from ethanol, Muto and co-workers identified bacteria from the genus *Neisseria* to have extremely high ADH activity and to be able to produce significant amounts of acetaldehyde (100-fold more than any other genera investigated) in the presence of ethanol in vitro (Table 2) [127]. The authors also found that the proportion of *Neisseria* species in the mouth increased when ingesting alcohol, suggesting that this bacteria genus may lead to high local acetaldehyde exposure and could possibly be involved in alcohol-related carcinogenesis in the oral cavity [127]. However, these strains are constituting only a small portion of the healthy oral microflora and therefore their contribution may be less relevant than that of high abundant and high acetaldehyde-producing strains [135]. Among the bacteria constituting the largest proportion of the normal oral microflora, those belonging to the genus *Streptococcus* have been investigated for ADH activity and acetaldehyde production (Table 2). Of the 16 *Streptococcus* strains investigated by Kurkivuori and co-workers, the clinical strain of *S. salivarius*, both clinical and culture collection strains of *S. intermedius*, and the culture collection strain of *S. mitis* showed significant ADH activity and produced high amounts of acetaldehyde when incubated with ethanol in vitro, underlying a possible role of Streptococci in ethanol-derived oral carcinogenesis [128]. 

The mechanisms by which streptococci produce a high amount of acetaldehyde from ethanol have been studied by constructing gene deletion mutants of *Streptococcus gordonii* V2016, which was identified to be able to produce high level of acetaldehyde from ethanol, and analysis of ADHs and ALDHs by zymograms [134]. Results showed that *S. gordonii* V2016 expressed three primary ADHs that were able to oxidize ethanol into acetaldehyde, but no ALDHs able to detoxify acetaldehyde into acetate. Further analysis of 19 additional strains of *S. gordonii*, *S. mitis*, *S. oralis*, *S. salivarius*, and *S. sanguinis* also revealed expression of up to three ADHs, but overall no detectable ALDHs, suggesting that the ability to produce acetaldehyde and increase its oral exposure results from a combination of multiple ADHs and absence of active ALDHs in most oral streptococci [134]. 

A step further was taken by Moritani and co-workers by assessing in vitro acetaldehyde production by 41 bacterial species belonging to 16 genera selected because they were predominant and prevalent in the saliva of 166 orally healthy subjects [133]. Among the considered species, all *Neisseria* as well as *Rothia mucilaginosa*, *Streptococcus mitis*, and *Prevotella histicola* were able to produce acetaldehyde from ethanol in quantities higher than 50 µM after incubation with 11 mM ethanol, a concentration that corresponds to 0.05% (*w/v*). For comparison, ethanol concentration in spirits varies from 30–90%, whereas that of wine and beer from 5–15%. Other species investigated possessed the ability to produce acetaldehyde, however, the amount of acetaldehyde produced was lower than 50 µM [133]. This study underlined how oral microbes of healthy subjects comprise several bacterial strains that are able to produce considerable amounts of acetaldehyde from ethanol, and emphasized the importance of knowledge of the individual salivary microbiota to possibly raise awareness on the acetaldehyde production, especially if high amounts of alcohol are frequently consumed [133]. 

The key role of microbially produced acetaldehyde in the oral cavity was also emphasized by Homann and co-workers, who demonstrated a significant reduction of in vivo produced acetaldehyde after use of an antiseptic agent, which was associated to a reduction in bacterial counts in saliva [25]. However, there was no correlation between individual total salivary bacterial counts and acetaldehyde level in saliva, indicating that a higher number of bacteria does not necessarily translate into a high level of acetaldehyde and that specific bacterial species may be responsible for most of the acetaldehyde produced in high acetaldehyde-producing individuals [25]. On the other hand, Yokoyama et al. found a correlation between oral bacterial and yeast counts and salivary acetaldehyde production, both of which decreased after three weeks of abstinence in Japanese alcohol-dependent men [32]. In another study, Homann and co-workers investigated factors responsible for altering the composition and quantities of the oral microflora and their influence in salivary acetaldehyde levels in 326 volunteers [3]. They identified smoking and heavy drinking as factors that increased microbial acetaldehyde production, even more if in combination, whereas poor dental health status did not correlate with an increased level of salivary acetaldehyde. Microbial analysis resulted in Gram positive aerobic bacteria and yeasts being associated with an increased production of acetaldehyde [3]. It is known that smoking changes the microbial composition and can increase infection with yeasts such as *Candida albicans* [136,137,138,139], an acetaldehyde-producing yeast [132], however, effects of heavy drinking on microbial composition is still unclear and will need further investigation. 

In order to investigate the relationship between oral microbiome and acetaldehyde production in cancer or diseased vs. healthy subjects, Marttila and co-workers investigated acetaldehyde production ability by the oral microbiome during in vitro ethanol incubation in microbial samples obtained from 30 oral squamous cell carcinoma patients, 30 patients with oral lichenoid disease, and 30 healthy control subjects [140]. They found that 68% of cultures were able to produce carcinogenic levels of acetaldehyde (>100 µM) when exposed to ethanol (22 mM), and that acetaldehyde production from smokers was significantly higher than that resulting from non-smokers. However, even if significant differences were found in the three groups regarding microbial counts, no differences in the acetaldehyde production were observed between samples from patients and healthy subjects [140]. On the same line, Alnuaimi and co-workers compared the ability of *Candida* isolates from oral cancer patients and matched oral healthy subjects to produce acetaldehyde [141]. Results revealed that *Candida* isolates producing higher amounts of acetaldehyde were more prevalent in patients with oral cancer than healthy volunteers, further underlying the possible role of *Candida* yeasts in alcohol-derived oral carcinogenesis as well as the importance of microbial strain identification in the evaluation of acetaldehyde exposure in the oral cavity [141]. 

There is enough evidence to prove that salivary acetaldehyde derives mostly from microbial metabolism, and that the composition of the oral microbiome is critical for assessing individual acetaldehyde exposure in the oral cavity. Therefore, not only bacterial counts, but rather a sequencing approach to identify microbial species should be used when investigating the microbial composition of the oral cavity, in order to identify if the predominant and prevalent bacterial species are known acetaldehyde-producers. More studies are needed to identify more bacteria and yeasts with acetaldehyde production capacity that may contribute to ethanol-derived acetaldehyde production in the oral cavity. Factors such as heavy drinking, smoking, and dental status can modify the composition of the oral microbiome and therefore indirectly influence the microbial production of acetaldehyde in the saliva to levels that are known to be mutagenic. All of these factors contribute to define different exposure scenarios and therefore have to be considered when assessing microbial-derived acetaldehyde exposure in the oral cavity. 

## 5. Acetaldehyde-Induced DNA Damage 

The toxicity of acetaldehyde is related to its reactivity. Acetaldehyde is an electrophilic molecule and it can react with nucleophilic centers in cellular constituents, including DNA, RNA, and proteins [142]. In particular, acetaldehyde carbonyl carbon reacts with DNA nucleosides generating DNA adducts. Experimental evidence reported that nucleobase reactivity with acetaldehyde decreases from deoxyguanosine (dG) to deoxyadenosine (dA) and deoxycitosine (dC) [143]. 

### 5.1. N^2^-Ethyl-dG: A Biomarker of Acetaldehyde-Induced DNA Damage

**Figure 2:**
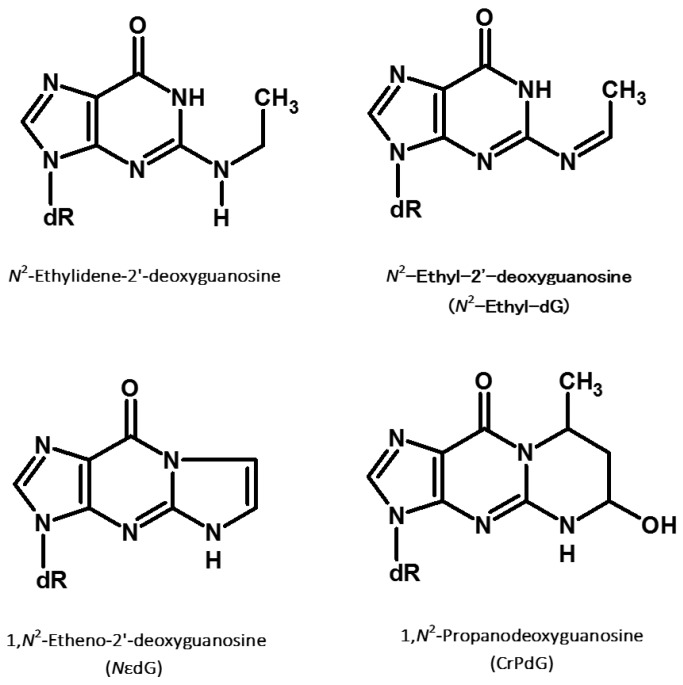
Previously studied acetaldehyde-derived DNA adducts.

The direct reaction product between acetaldehyde and the nucleoside dG, *N*^2^-ethylidene-dG (Figure 2), was originally identified by Vaca and colleagues [143]. This DNA modification is an instable Schiff base (half-life of 24 h at 37 °C in vivo), which can be stabilized through reduction with NaBH_3_CN resulting in *N*^2^-ethyl-dG (Figure 2) [142]. *N*^2^-ethyl-dG is also found in vivo, potentially due to the reducing action on *N*^2^-ethilydene-dG of GSH or vitamin C [143].

Vaca and colleagues first developed a ^32^P-postlabelling method that was able to detect an increase of *N*^2^-ethyl-dG in liver DNA of rats given 10% ethanol in water (1 adduct per 10^8^ nucleosides) [143]. This study was followed by several other studies focused on the detection of *N*^2^-ethyl-dG in vivo. Robust and sensitive analytical methods using isotope dilution mass spectrometry were developed in order to quantify *N*^2^-ethyl-dG in complex biological samples [27,28]. It was found that the adduct is already relatively abundant in human liver DNA in levels of 1 adducts per 10^7^ nucleotides [144]. The presence of the adduct could result from reaction of DNA with endogenous acetaldehyde produced through metabolism of threonine, alanine, and deoxyribose phosphate [145], or as a result of oxidative stress, possibly due to the inhibition of acetaldehyde oxidation [146]. On the other hand, increased levels of *N*^2^-ethyl-dG were found in the oral cavity, liver, stomach, brain, and esophagus of animals exposed to alcohol [18,147,148,149,150], and in human blood and oral cells of subjects ingesting alcohol [27,28]. An increase in *N*^2^-ethyl-dG was also observed in blood of ALDH2-deficient drinkers [66]. This study reported a significant increase of *N*^2^-ethyl-dG in ALDH2-deficient drinkers and supported the classification of acetaldehyde related to alcohol consumption as carcinogenic to humans [66].

The mechanistic role of *N*^2^-ethyl-dG in carcinogenesis still remains unclear, but is possibly related to the DNA damage accumulating in the body due to inefficient DNA repair [148,151]. The absence of repair and accumulation of *N*^2^-ethyl-dG adducts was observed in ALDH2-knockout mice treated with ethanol, where an increase of *N*^2^-ethyl-dG was observed over time during ethanol administration [148]. In addition, the study of nucleotide incorporation opposite *N*^2^-ethyl-dG by DNA polymerases reported a different behavior in bacterial vs. mammalian cells. A study by Shibutani and colleagues highlighted that both dC and dG can be incorporated opposite *N*^2^-ethyl-dG by *Escherichia Coli* DNA polymerase I in vitro [152]. Differently, in mammalian cells DNA polymerases exert a diverse action on the modification. *N*^2^-ethyl-dG strongly blocks replication by DNA polymerase α, but is bypassed by DNA polymerase η by incorporation of dC opposite the lesion [153]. On the other hand, Pence and colleagues reported that DNA polymerase ι can bypass *N*^2^-ethyl-dG by changing its position into a *syn* configuration [154]. Finally, another study demonstrated that *N*^2^-ethyl-dG in DNA exerts its principal biological activity by blocking translesion DNA synthesis in human cells, resulting in either failure of replication or frameshift deletion mutations [155]. On the other hand, *N*^2^-ethyl-dGTP incorporation by DNA polymerases was only studied in the presence of the replicative DNA polymerase δ, which only incorporated *N*^2^-ethyl-dGTP opposite the dC template, the correct base [156]. However, all these studies have been performed on the stable reduced analog of *N*^2^-ethylidene-dG and how these results translate to the original adduct remains to be elucidated.

For what concerns the oral cavity, the hypothesis that ethanol-derived acetaldehyde exposure may result in DNA damage was supported by recent results showing the presence of *N*^2^-ethyl-dG in oral tissues and cells of non-human primates and humans exposed to alcohol [18,28]. In a first study, Balbo et al. measured *N*^2^-ethyl-dG in DNA derived from human oral exfoliating cells from ten healthy non-smokers at several time points after consumption of increasing doses of alcohol [28]. Results showed a 100-fold increase in *N*^2^-ethyl-dG compared to baseline values within four hours after each dose and in a dose response manner in all subjects. Despite the small number of participants, the results were significant even after the first dose and indicated a direct link between alcohol consumption and DNA damage as well as provided the first evidence of the kinetics of formation and increase *N*^2^-ethyl-dG in the oral cavity of humans exposed to alcohol [28]. 

In a second study, *N*^2^-ethyl-dG was quantified in DNA from oral, esophageal, and mammary gland tissues of Rhesus monkeys exposed to alcohol drinking over their lifetime [18]. *N*^2^-ethyl-dG levels in the oral mucosa, but not in the esophageal mucosa and mammary glands of female animals, were significantly higher in the exposed animals compared to unexposed controls, supporting the previous findings of acetaldehyde being able to damage the DNA of the oral mucosa if exposed to alcohol, as well as the importance of evaluating oral ethanol metabolism independently form liver ethanol metabolism when investigating alcohol as oral cancer risk factor [18]. These results were crucial also because they demonstrated measurement and presence of this DNA modification as a consequence of alcohol-derived acetaldehyde at the oral tissue level and not only in superficial exfoliating cells, which represent the first layers of cells covering the oral cavity and therefore those that are directly exposed to alcohol. 

In summary, the mutagenic role of *N*^2^-ethyl-dG and its carcinogenicity still need to be assessed. In particular, it is yet unclear how *N*^2^-ethyl-dG levels are affected by repair mechanisms. However, the development of validated, quantitative, rigorous, and sensitive analytical methods for *N*^2^-ethyl-dG, and their application in epidemiological studies make this adduct an excellent biomarker for studying acetaldehyde derived DNA damage.

### 5.2. Other Acetaldehyde-Induced DNA Adducts

The reaction between two molecules of acetaldehyde and DNA leads to the formation of another class of adducts: crotonaldehyde-derived propano-dGs (CrPdGs, Figure 2). Garcia and colleagues postulated the formation of CrPdGs to result from two subsequent reactions between acetaldehyde and DNA instead of by the reaction of DNA and crotonaldehyde, based on the absence of crotonaldehyde in their cell media [157]. However, another possible mechanism of CrPdG formation could involve the condensation of two molecules of acetaldehyde into crotonaldehyde followed by reaction of crotonaldehyde with DNA [158]. The ultimate formation mechanisms have not been clarified yet. 

CrPdGs are, as *N*^2^-ethyl-dG, Schiff bases on the same amino group of dG. Differently from *N*^2^-ethyl-dG, studies demonstrated that CrPdGs may be repaired and not accumulated [148]. However, despite being repaired, CrPdGs adducts have multiple biological effects. They promote DNA miscoding in human cells by G→T transversion mutations and they can inhibit DNA synthesis [159]. Moreover, this class of adducts can change conformation into a ring opening form, leading to the formation of another aldehyde carbonyl center, which can react with another dG creating a DNA interstrand crosslink that could inhibit DNA replication [151].

Etheno-dG (*N*εdG, Figure 2) is considered by Brooks and Zakhari a secondary acetaldehyde adduct due to the fact that it cannot be formed by a direct interaction between acetaldehyde and DNA. However, Garcia and colleagues found a high concentration of *N*εdG in human cells after acetaldehyde exposure [157]. This evidence highlighted that *N*εdG could result from a dose-dependent increase in lipid peroxidation induced by acetaldehyde exposure, which leads to the formation of *N*εdG by interaction of DNA and lipid peroxides [157]. *N*εdG can lead to a wide spectrum of biological responses. It can block replicative polymerases δ, meanwhile different translesion polymerases can bypass the DNA modification leading to mutagenic consequences [160]. In summary, CrPdGs and *N*εdG are two other classes of adducts found to be directly or indirectly related with acetaldehyde exposure. They are less studied than *N*^2^-ethyl-dG and their biological relevance in carcinogenesis is not completely assessed yet. 

Overall, information is available on the levels of *N*^2^-ethyl-dG in several organs or tissues, as well as information about its bypass by DNA polymerases and repair. Studies about levels and bypass for the other two DNA adducts resulting from exposure to acetaldehyde are scarce. In addition, knowledge regarding their repair in cells is lacking. Information about the efficiency of repair of these adducts could be useful for assessing their biological significance and could help provide evidence of their role in acetaldehyde-related carcinogenesis. Furthermore, these studies focused on the reaction of acetaldehyde and guanine, and studies evaluating adducts induced by reaction with other nucleosides that could have biological significance are missing. 

## 6. Future Developments and Challenges 

The oral cavity is a complex environment characterized by a variety of endogenous and exogenous exposures, and inhabited by millions of microbes. Due to its complexity, assessing exposures in the oral cavity is a challenging task, and requires simultaneous consideration of as many factors as possible, which may be involved or modulate the levels of the chemical/biomarker to be measured. In the case of assessing ethanol-derived acetaldehyde exposure in the oral cavity, the principal factors to be considered are those who could influence ethanol metabolism and acetaldehyde levels such as genetic polymorphisms (ADH, ALDH, and CYP2E1), oral hygiene status, nutritional, lifestyle and environmental factors (e.g., type of alcoholic beverage, smoking, and drinking habits), and metabolism of individual oral mucosa cells, salivary glands, and microbiome. 

In the past, the influence of these factors on assessing salivary ethanol-derived acetaldehyde exposure has been mostly only individually considered, resulting in many studies distributed over 20 years that investigated how one or just a few factors modulated acetaldehyde levels in the oral cavity. The development of high-throughput screening and highly sensitive techniques in the field of analytical chemistry, as well as advancements in genome sequencing for the characterization of microbial species in the past decade may be of great advantage in performing a comprehensive evaluation of the contribution of these factors to acetaldehyde exposure in the oral cavity. Nevertheless, the output of such an exposure assessment will result in the combined contribution of these factors, and will account also for synergistic effects, such as those resulting from a combination of alcohol drinking and tobacco smoking [37].

Of great importance when evaluating alcohol-derived acetaldehyde exposure in the oral cavity is the contribution of the oral microbiome. In fact, it has been shown that most of the ethanol-derived acetaldehyde in the oral cavity results from microbial metabolism. Several yeasts and bacterial strains with ADH capability and able to produce acetaldehyde have been previously identified (Table 2), however, it is not known how many other microbial strains constituting a normal or diseased oral cavity are able to metabolize ethanol into acetaldehyde, and to which extent. Therefore, more research is needed to identify microbial strains with acetaldehyde-producing capabilities and to assess their prevalence in the individual oral microbiome in order to identify the strains that mostly contribute to acetaldehyde production in the oral cavity. For these reasons, sequencing approaches that result in the characterization of the microbiome at the strain level should be preferred to identify those strains that are already known to be able to produce acetaldehyde, and to select relevant strains that are either more abundant or related to one of the known producing strains. Selected strains can then be investigated for their acetaldehyde-producing capacity in vitro, and for their expression and activity of acetaldehyde-metabolizing enzymes.

A strategy for evaluating acetaldehyde exposure in the oral cavity that would allow simultaneous consideration of all the factors influencing acetaldehyde production and levels involves the quantitation of acetaldehyde-derived DNA adducts. In fact, an increase in the level of acetaldehyde in the oral cavity is only the first step in the manifestation of ethanol toxicity, and measuring acetaldehyde-induced DNA adducts would provide results of a further step in the evaluation of the role of acetaldehyde in oral cancer development, since acetaldehyde-derived DNA modifications may lead to incorrect, incomplete or inhibited repair and ultimately result in mutations. Additionally, DNA adducts as biomarkers of acetaldehyde exposure could be used to set cut-off levels to derive the risk of developing cancer of the oral cavity and identify susceptible individuals, as well as evaluate the implementation of preventive strategies. 

*N*^2^-ethyl-dG, the most abundant acetaldehyde-induced DNA adduct, has been successfully quantified in DNA isolated from the oral cells of healthy non-smokers and smokers [28], and from tissue samples of Rhesus monkeys exposed to alcohol [18]. Recently, Balbo and co-workers developed several DNA adductomic approaches for the identification and relative quantitation of several adducts resulting from many types of exposures in biological samples [161,162,163,164]. Such an approach is planned to be used for measuring not only *N*^2^-ethyl-dG, but also other DNA adducts induced by acetaldehyde, to potentially identify new acetaldehyde-derived DNA adducts to be targeted during the overall exposure assessment. 

In conclusion, there is enough evidence to support the hypothesis that alcohol metabolism in the oral cavity has to be evaluated as an independent cancer risk factor and not as part of alcohol liver metabolism. Assessment of ethanol-derived acetaldehyde exposure in the oral cavity is a challenging task due to the multiple factors, which can play a role in acetaldehyde production from alcohol consumption. The identification, validation, and use of biomarkers able to evaluate the influence of each factor separately or in combination is necessary to provide a solid base for investigating the role of alcohol-derived acetaldehyde in increasing the risk of UADT cancers. 

## Figures and Tables

**Table 1:** Acetaldehyde levels measured in alcoholic beverages.

Type of Alcoholic Beverage	Subcategory (If Present)	Acetaldehyde (µM) ^1^	*n* ^2^	Ref.
Apple wine/cider		1123 ± 932	11	[100]
		2529	1	[23]
Beer		140	1	[22]
		120	1	[23]
		205 ± 150	364	[100]
		233 ± 281	6	[101]
		172 ± 67	3	[102]
		192	12	[40] ^8^
Fortified wines		586	7	[40] ^8^
		2686 ± 2728	133	[100]
		2231 ± 2450	53	[100]
	Cherry spirit	8522	1	[23]
	Port	1909 ± 3306	27	[100]
	Sherry	2583	1	[23]
		3537 ± 2482	53	[100]
Liquors and spirits		1251 ± 1155	12	[101]
		1541 ± 2344	834	[100]
		972	61	[40] ^8^
	Bacanora ^3^	7711 ± 5061	13	[100]
	Brandy/Cognac	1704 ± 1096	82	[100]
	Cachaça ^4^	1149 ± 491	21	[100]
	Calvados	1781 ± 861	25	[101]
		600	1	[22]
		753 ± 342	2	[23]
		870 ± 334	27	[100]
	Chinese spirits	7419 ± 3955	30	[100]
	Fruit-based	1953 ± 2704	315	[100]
		1414	17	[40] ^8^
	Gin	21	3	[40] ^8^
	Grape mark spirit	12,903 ± 2697	4	[23]
	Grappa ^5^	11,327	13	[40] ^8^
	Herb and spice-based	638	11	[40] ^8^
	Mezcal	2103 ± 2024	10	[100]
	Rum	3110	3	[40] ^8^
		403 ± 321	38	[100]
	Sake	717 ± 359	5	[102]
	Shochu ^6^	600	1	[22]
	Sotol ^7^	1876 ± 1346	16	[100]
	Tequila	530	1	[23]
		1371 ± 1960	70	[100]
	Vodka	48	3	[40] ^8^
		61 ± 70	72	[100]
	Whiskey	1746	3	[40] ^8^
		1410 ± 715	3	[102]
		627 ± 448	37	[100]
Wine		275 ± 236	6	[101]
		474	1	[23]
		773 ± 760	213	[100]
		1140 ± 308	3	[102]
		1544	60	[40] ^8^
	Red wine	1267	21	[40] ^8^
		250	1	[22]
	Rose wine	1855	3	[40] ^8^
	Sparkling wine	2792	15	[40] ^8^
	White wine	1521	21	[40] ^8^
Pure alcohol		56	1	[40] ^8^

^1^ Amounts (ppm, mg/L, etc.) were converted to concentrations and reported in µM. They correspond to the average and standard deviation resulting from measurement of replicate samples; ^2^ *n* corresponds to the number of samples used to derive average and standard deviation values; ^3^ Bacanora is an agave-derived liquor made in the Mexican state of Sonora. ^4^ Grappa is a grape-based alcoholic beverage of Italian origin that contains 35% to 60% alcohol by volume; ^5^ Cachaça is a distilled spirit made from fermented sugarcane juice; ^6^ Shochu is a Japanese distilled beverage with less than 45% alcohol by volume typically distilled from rice, barley, sweet potatoes, buckwheat, or brown sugar; ^7^ Sotol is a distilled spirit made from the *Dasylirion wheeleri, Asparagaceae*, a plant that grows in Northern Mexico, New Mexico, west Texas, and the Texas Hill Country; ^8^ Values reported from reference [40] correspond to the medians calculated from replicate samples.

**Table 2:** Oral microbial strains possessing ADH activity and able to metabolize ethanol or glucose into acetaldehyde in vitro.

Type	Species	Isolate	In Vitro Acetaldehyde Production from Ethanol	In Vitro Acetaldehyde Production from Glucose	Ref.
Yeasts	*Candida albicans*	Culture and subject strains	157.43 ± 1.57 µM (ATCC 90029), 247.9 ± 4.2 µM (APECED), 280.2 ± 11.8 µM (cancer), 299.1 ± 12.7 (controls) µM	38.02 ± 2.06 µM (ATCC 90029), 53.5 ± 2.3 µM (APECED), 33.7 ± 3.5 µM (cancer), 34.6 ± 1.9 µM (controls)	[129]
		Subject strains	-	619.4 µM (APECED), 716.6 µM (cancer), 654.0 µM (controls)	[130]
		ATCC90029	235.1 ± 2.8 µM	18.7 ± 2.9 µM	[131]
		Subject strains	73.7 ± 55.4 µM (high-producing saliva), 43.2 ± 22.3 µM (low-producing saliva)	-	[132]
	*C. dubliniensis*	Isolates from culture collection strains	139.0 ± 7.1 µM	4.6 ± 1.1 µM	[131]
	*C. glabrata*	Isolates from culture collection strains	185.6 ± 8.6 µM	5.4 ± 1.1 µM	[131]
		TIMM 5512	-	83.1 ± 24.8 µM	[131]
	*C. guilliermondii*	Isolates from culture collection strains	190.2 ± 12.4 µM	7.3 ± 1.7 µM	[131]
	*C. kefyr*	TIMM 0298	299.3 ± 80.9 µM	80.1 ± 16.8 µM	[133]
	*C. krusei*	Isolates from culture collection strains	54.6 ± 2.9 µM	2.6 ± 0.9 µM	[131]
	*C. parapsilosis*	Isolates from culture collection strains	243.3 ± 8.8 µM	45.2 ± 2.1 µM	[131]
	*C. tropicalis*	isolates from culture collection strains	252.3 ± 14.9 µM	81.8 ± 2.9 µM	[131]
		TIMM 0313	248.9 ± 49.9 µM	-	[133]
Bacteria	*Neisseria flava*	ATCC 14221	94.4 ± 2.1 µM	-	[133]
	*N. flavescens*	ATCC 13120	168.4 ± 8.6 µM	-	[133]
	*N. mucosa*	ATCC 19695	272.8 ± 65.5 µM	-	[133]
	*N. sicca*	ATCC29256	174.9 ± 39.4 nmol/min/10^11^ CFU	-	[127]
	*N. subflava*	9903683	23,050.4 ± 1624.7 nmol/min/10^11^ CFU	-	[127]
	*Prevotella histicola*	JCM 15637	53.2 ± 10.1 µM	-	[133]
	*Rothia mucilaginosa*	JCM 10910	95.9 ± 5.9 µM	-	[133]
	*Streptococcus australis*		-	-	[133]
	*S. gordonii*		-	-	[134]
	*S. intermedius*		-	-	[128]
	*S. mitis*	JCM 12971	90.2 ± 31.3 µM	-	[133]
			-	-	[128]
	*S. oralis*		-	-	[134]
	*S. parasanguis*		-	-	[133]
	*S. salivarius*	T-42104	135.0-426.3 µM	-	[128]
	*S. sanguinis*		-	-	[134]
