# Correction: Alessia Stornetta et al. Alcohol-Derived Acetaldehyde Exposure in the Oral Cavity. *Cancers* 2018, *10*, 20

**DOI:** 10.3390/cancers10030074

**Published:** 2018-03-16

**Authors:** Alessia Stornetta, Valeria Guidolin, Silvia Balbo

**Affiliations:** 1 Masonic Cancer Center, University of Minnesota, Minneapolis, MN 55455, USA; storn005@umn.edu (A.S.); guido019@umn.edu (V.G.); 2 Division of Environmental Health Sciences, University of Minnesota, Minneapolis, MN 55455, USA

The authors would like to make a correction to their published paper [1].

There was a mistake in the original version of the article in Figure 2. The two structures at the top of the figure were inverted and the bottom right structure (1,*N*^2^-Propano-2′-deoxyguanosine) contained a mistake. The authors have corrected the errors as shown in the figure below. The figure legend does not need to be changed.

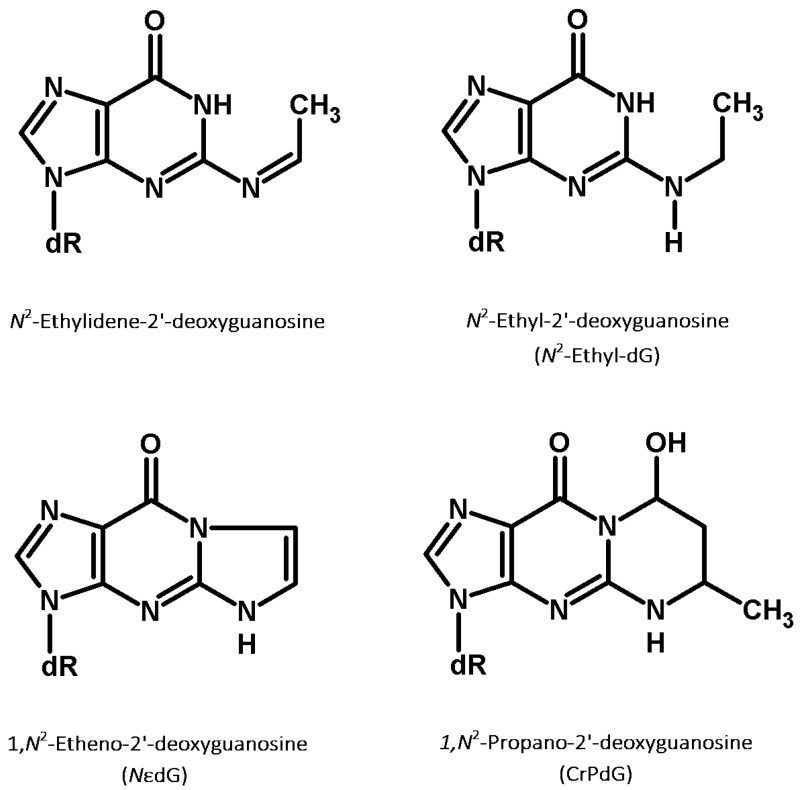



In addition, there was a mistake on page 4 in the paragraph describing reference 34. The number of subjects evaluated in this study is 526 cancer-free and 159 cancer subjects, and not 34 as specified in the previous version of the manuscript. In addition, reference 34 investigated ADH2 and not ADH1B. The authors have corrected the errors as shown in the paragraph below:

“The relationship between ADH2 polymorphism and head and neck cancers was evaluated in a case-control study, in which the DNA of Japanese alcohol-dependent men (526 cancer-free, and 159 who had one or more cancers of the aerodigestive tract) was genotyped [34]. It was found that the presence of the ADH2*1/2*1 genotype significantly increased the risk for oropharyngolaryngeal cancer, with an overall 6.68 odds ratio. The study also calculated individual odds ratios of 5.48 and 6.57 for oropharyngeal and laryngeal cancers, respectively. Meanwhile, the coexistence of ADH2*1/2*1 and ALDH2*1/2*2 showed to have a synergistic effect on the risk of oropharyngolaryngeal cancer, with an overall odds ratio of 121.77 [34]”.

The rest of the manuscript does not need to be changed. The authors would like to apologize for any inconvenience caused. The manuscript will be updated and the original will remain available on the article webpage.
